# AllerCatPro—prediction of protein allergenicity potential from the protein sequence

**DOI:** 10.1093/bioinformatics/btz029

**Published:** 2019-01-18

**Authors:** Sebastian Maurer-Stroh, Nora L Krutz, Petra S Kern, Vithiagaran Gunalan, Minh N Nguyen, Vachiranee Limviphuvadh, Frank Eisenhaber, G Frank Gerberick

**Affiliations:** 1 Biomolecular Function Discovery Division, Bioinformatics Institute, Agency for Science, Technology and Research, Singapore; 2 Department of Biological Sciences, National University of Singapore, Singapore; 3 The Procter & Gamble Services Company, Strombeek-Bever, Belgium; 4 The Procter and Gamble Company, Mason, OH, USA

## Abstract

**Motivation:**

Due to the risk of inducing an immediate Type I (IgE-mediated) allergic response, proteins intended for use in consumer products must be investigated for their allergenic potential before introduction into the marketplace. The FAO/WHO guidelines for computational assessment of allergenic potential of proteins based on short peptide hits and linear sequence window identity thresholds misclassify many proteins as allergens.

**Results:**

We developed AllerCatPro which predicts the allergenic potential of proteins based on similarity of their 3D protein structure as well as their amino acid sequence compared with a data set of known protein allergens comprising of 4180 unique allergenic protein sequences derived from the union of the major databases Food Allergy Research and Resource Program, Comprehensive Protein Allergen Resource, WHO/International Union of Immunological Societies, UniProtKB and Allergome. We extended the hexamer hit rule by removing peptides with high probability of random occurrence measured by sequence entropy as well as requiring 3 or more hexamer hits consistent with natural linear epitope patterns in known allergens. This is complemented with a Gluten-like repeat pattern detection. We also switched from a linear sequence window similarity to a B-cell epitope-like 3D surface similarity window which became possible through extensive 3D structure modeling covering the majority (74%) of allergens. In case no structure similarity is found, the decision workflow reverts to the old linear sequence window rule. The overall accuracy of AllerCatPro is 84% compared with other current methods which range from 51 to 73%. Both the FAO/WHO rules and AllerCatPro achieve highest sensitivity but AllerCatPro provides a 37-fold increase in specificity.

**Availability and implementation:**

https://allercatpro.bii.a-star.edu.sg/

**Supplementary information:**

Supplementary data are available at *Bioinformatics* online.

## 1 Introduction

Protein allergens contain immunogenic and antigenic structures that can lead to an immunoglobulin (Ig) E-mediated respiratory (Type I) allergy. IgE-sensitization towards proteins is frequently recognized in the context of aeroallergens (e.g. pollen) and food allergens (Pawankar *et al.*, 2013) as well as personal care products (Fukutomi *et al.*, 2014; Troyano *et al.*, 2011). The assessment of the allergenic potential of novel proteins remains a challenge since there is no generally accepted, validated and broadly applicable method available (Verhoeckx *et al.*, 2016). The current approach relies on the guidance of allergenicity assessment for genetically modified plant foods recommended by FAO/WHO (2001), which is based on single hexamer peptide hits and sequence identity thresholds to known allergens. However, this similarity approach leads to many wrongly classified proteins as potentially allergenic (Stadler and Stadler, 2003), including up to 90% of all human proteins (Supplementary Fig. S1).

Many factors are known to contribute to protein allergenicity (Huby *et al.*, 2000), including protein stability, cleavage sites, post-translational modifications and physico-chemical properties. However, allergenic proteins need to be recognized by T and B cells to trigger the development of protein-specific IgE and/or they need to react with IgE on basophiles or mast cells to trigger the elicitation of an IgE-mediated allergic reaction. The basis for this specific immune recognition of the protein is its 3D structure and its amino acid sequence.

Here, we present a new model to predict the protein allergenicity potential starting from the protein sequence. We first gathered all available and reliable protein sequences associated with allergenicity (further abbreviated as ‘known allergens’) and analyzed these protein sequences and their corresponding 3D structures to identify and characterize features related to allergenicity and then combined these features with a biophysical model built on the union of available data sets to form one unique and comprehensive data set.

## 2 Materials and methods

### 2.1 Merged database

The five major databases of known allergens online were accessed and sequences were retrieved directly or via accessions through the respective databases at NCBI or UniProt. Next, cd-hit (Li and Godzik, 2006) was used to create non-redundant subsets with the detailed resulting numbers of unique proteins for each database in Table 1.

**Table 1.** Source databases for known allergenic proteins

Database	Weblink	Date accessed	No. unique proteins
IUIS	http://www.allergen.org/	September 2017	779
UniProtKB	https://www.uniprot.org/	September 2017	704
(key word: ‘allergen’)			
Allergome	http://www.allergome.org/	September–November 2017	3157^a^
COMPARE	http://comparedatabase.org/	February 2018	2022
FARRP	http://allergenonline.org/	March 2018	2069

a With evidence from functional tests or epidemiology.

In the case of Allergome, individual entries were accessed online and sequences retrieved with the additional criterion that the evidence of allergenicity includes at least one strong experimental test (without counting non-functional tests) or epidemiological support. Supplementary Table S1 lists the accessions of the entries considered from the respective databases.

### 2.2 k-mer hit criterion

First, a query protein was split into its respective 6-mers and those of low complexity as defined by a sequence entropy < 0.34 (log2-based bit score) and those with ambiguous amino acids (BJOUXZ) were removed. Then the remaining query 6-mers were compared with the 6-mer database derived from our database of known allergens. A hit to a known allergen is found, if at least three different 6-mers are shared between the known allergen and the query protein.

### 2.3 Gluten-like Q-repeat fingerprint score

From the Food Allergy Research and Resource Program (FARRP) AllergenOnline database, 1013 ‘Celiac disease peptides’ were downloaded in March 2018. The smallest size of those peptides is nine residues, which are in agreement with most major histocompatibility complex Classes I and II core-binding regions. The amino acid frequencies were calculated for every 9-mer window within the peptides and a composition fingerprint score was derived by using a log odd ratio of the frequency in the ‘Celiac 9-mer’ windows divided by a background database frequency (UniProtKB used here). This log odd score is used to score all 9-mers in a query protein and if the score for a 9-mer is within one standard deviation of the average of the FARRP ‘Celiac disease peptides’, it triggers a hit as Gluten-like Q-repeat.

### 2.4 3D structure/model database

Cd-hit (Li and Godzik, 2006) was used to cluster the known allergens into groups of 70% or more sequence identity. We next used BLAST (Altschul *et al.*, 1997) and HHpred (Zimmermann *et al.*, 2018) against PDB (Burley *et al.*, 2017) to find templates for homology modeling for the ∼1200 representatives. Approximately 900 models were created using MODELLER (Webb and Sali, 2017) in two steps. The modeling process was performed in two steps. First, dynamic programming-based structural alignment between query and template was performed by using the salign class of MODELLER and then 100 structural models were built and the discrete optimized protein energy (DOPE) score of each model was calculated and the one with the lowest energy was selected for Step 2, the loop refinement. Using the loop model class of MODELLER, 200 models with refined loops were built and the one with the lowest DOPE score was selected as the final model. Next, we further evaluate model quality visually and with ProQ2 (Ray *et al.*, 2012) requiring quality thresholds of LGscore > 1.5 and MaxSub > 0.1. This resulted in 713 representative protein structures/models. To ensure that the resulting models are consistent with optimal protein geometry, we next use YASARA (Krieger and Vriend, 2014) to calculate Z-scores for deviation from normality of angles, bonds, dihedrals (Ramachandran plot) and planarity relative to the AMBER-FB15 force field (Wang *et al.*, 2017). This identified 91 suboptimal models with Z-score < −2. Using the YASARA energy minimization protocol (Krieger *et al.*, 2009) based on short simulated annealing molecular dynamics simulations with the AMBER-FB15 force field, we corrected the 91 models. Supplementary Table S3 lists details on template similarity and model quality.

### 2.5 Sequence to 3D epitope mapping

For every structure a table of epitope definitions was created following this procedure: First, all surface accessible residues were identified with YASARA (distance to solvent accessible surface <2.55 Å, empirically derived threshold that was consistent with the binding interface of known protein-antibody complexes), then each of the surface residues were taken as the hypothetical center of an epitope and all other surface residues that are within 12 Å distance from the center residue were included. This distance was chosen to match the binding interface size (Dall’antonia *et al.*, 2014) seen in representative complexes of IgE antibodies with allergens. A minimum epitope size of at least 13 residues is further required. The procedure is implemented as custom Yanaconda macro script in YASARA (Krieger and Vriend, 2014).

For the sequence to epitope mapping, the query protein is searched with BLASTP (Altschul *et al.*, 1997) against our 3 D structure/model database (E-value < 0.001). To compare the query protein to the closest known allergen in context of the 3D structure an additional BLASTP search (E-value < 0.001) is run with the query protein against our database of known allergens. Next, MAFFT (Katoh and Standley, 2014) is used with L-INS-I settings to create a multiple sequence alignment of the three sequences: query, best 3D hit and best allergen hit. The aligned residues of query and allergen with the structure are then assigned to the respective epitopes using the epitope definition table described above. Finally, a loop over all epitopes comparing the identity of epitope residues between query and allergen allows determination of the epitope with highest identity. In case of equal identity values, the larger epitope is considered. This procedure is implemented as custom Perl scripts.

## 3 Results

### 3.1 Comprehensive database of known allergens

Various *in silico* databases of protein allergens were reviewed to gather available allergenicity information on characterized proteins (Table 1) to help identify the allergenic potential of novel proteins. The most comprehensive database is Allergome (http://www.allergome.org/) which provides annotation details for each entry to characterize the degree of allergenicity based on available data from literature and from the ‘Real Time Monitoring of IgE-sensitization’ database, which provides data from any contributor willing to share data. The most popular database for assessment of food allergen proteins is the Allergen Online database from the FARRP (https://farrp.unl.edu/resources/farrp-databases). This database contains information collected and evaluated by a peer review panel of scientists and clinicians comparing peer reviewed publications following pre-determined guidelines. The most recent Comprehensive Protein Allergen Resource (COMPARE, http://comparedatabase.org/database/) by the Health and Environment Science Institute comprises protein entries which result from an algorithm combined with a review of the corresponding literature and a final decision made by independent allergy experts. The UniProtKB, although not specialized on allergens alone, is one of the most established sources for general protein annotations. It is based on a combination of manual curation and annotation by close similarity and uses the controlled keyword “Allergen” to attribute allergenic potential to proteins. One of the most stringent databases regarding criteria that need to be fulfilled to consider a protein as allergenic is organized by the Allergen Nomenclature Sub-Committee under the auspices of the WHO and the International Union of Immunological Societies (IUIS, http://www.allergen.org/). A protein is considered as allergenic if protein-specific IgE-reactivity was demonstrated with sera from at least five patients allergic to the protein source and, moreover, the protein has been characterized in accordance to given WHO/IUIS criteria (Pomes *et al.*, 2018).

**Fig. 1.**
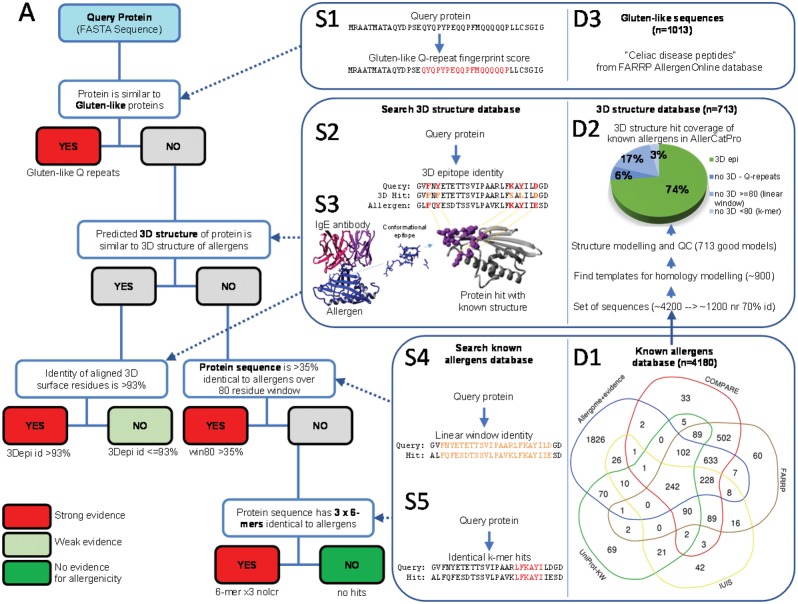
AllerCatPro workflow, search methods and databases. **(A)** Decision workflow of AllerCatPro from the query protein to the results of either strong, weak or no evidence for allergenic potential. (S1–S5) Search methods utilized at different stages of the workflow. (D1–D3) Databases created and used for the searches in the workflow

Using this collection of major databases with most including various degrees of expert curation, we systematically compared their data overlap using 100% sequence identity as criterion to determine shared and unique proteins. There is a strong consensus between individual databases featuring only 33–69 protein entries unique to only one database except for Allergome which contains 1826 unique sequences (Fig. 1, D1). The total number of unique entries merged from the 5 databases comprises 4180 proteins with good support for allergenic potential, which we then use as known allergens for our computational workflow described here.

### 3.2 Improved k-mer matches with known allergens avoiding random hits

One of the traditional criteria that have been used for allergenicity assessment of genetically modified plant foods by experts for the FAO/WHO (2001) is the six-amino acid rule: A protein matching a k-mer of six amino acids in length with a known allergen should be further evaluated for potential allergenicity. The statistical distribution of k-mer matches between two sequences (Lippert *et al.*, 2002) and for database searches (Tan *et al.*, 2012) is well studied. In short, there is a critical k-mer length for a given database (depending on size and redundancy within). Below the critical k-mer length, random hits increase. Above the critical k-mer length, the k-mer becomes specific only for the respective protein, unless the k-mer represents a simple low complexity sequence repeat, which in turn produces random hits (Tan *et al.*, 2012). In the case of allergen databases, the number of known allergens has grown dramatically in the last decade and the probability of finding a random 6-mer hit to an allergen, for example in the human proteome, is so high that 90% of human proteins would be classified as allergens with this rule (Supplementary Fig. S1).

A simple temporary solution is to increase the k-mer length, for example to at least eight, as suggested by Goodman (2006) and Hileman *et al.* (2002). Although the general value of k-mer-based hits are frequently questioned (Herman *et al.*, 2009), one reason for their introduction was to evaluate potential immunogenic cross-reactivity which can occur at the T-cell epitope level (Westernberg *et al.*, 2016). AllerCatPro strikes a conservative balance between the need for safety and the practicality of avoiding random hits by using a statistically informed k-mer hit criteria. First, low complexity sequence motifs are detected and filtered out with simple sequence entropy measures (Wootton and Federhen, 1996). Second, instead of increasing the k-mer length a minimum number of k-mer hits within the same protein is required. For example, for a k-mer of length six, three consecutive hits (shifted by only one position) exactly fit into a sequence of eight residues and, hence, are a more flexible form of 8-mer matches that also allow matches to more relaxed patterns of homology seen in protein sequences. This approach is also in agreement with the rationale of similarity to T-cell epitopes, where there is usually not a single long epitope in a protein sequence but multiple short ones (in terms of the recognized core region) (Huby *et al.*, 2000; Jahn-Schmid *et al.*, 2005; O’Brien *et al.*, 1995; Oseroff *et al.*, 2012; Prickett *et al.*, 2015).

**Fig. 2.**
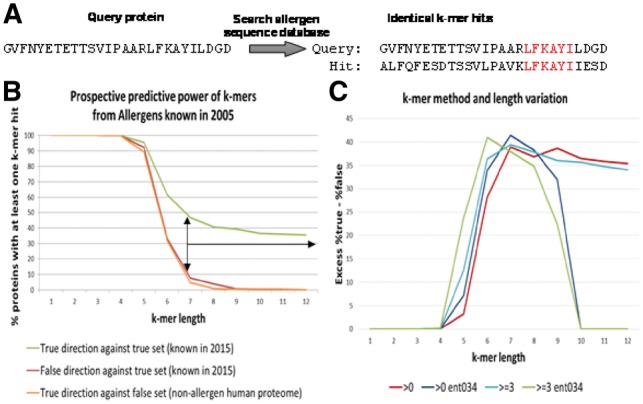
Prediction of protein sequence similarity towards protein allergens by the k-mer method. Screening for similarity between a query protein sequence and a sequence in the allergen database is based on identical k-mer hits **(A)**. Evaluation of an appropriate k-mer length based on the predictive power of different k-mer lengths by using the UniProt database of known allergens from 2005 to predict all allergens known in 2015 **(B)**. Differences in the excess of percent true minus false positives depending on the k-mer length and entropy degree (ent034 = entropy bit score > 0.34) **(C)**

In order to evaluate the best combination of the k-mer length and parameters discussed earlier, the prospective predictive power of different k-mer lengths was estimated using the UniProt database of known allergens from 2005 to predict all allergens known in 2015 following the rule of having at least one k-mer hit to a known allergen (Fig. 2B). To estimate the rate of false positives two ‘false’ control sets were used that should not be natural allergens and not detected as such. The first is the sequence reversal (same protein sequence in false direction) of the true set which is a perfect non-sensical copy maintaining number and length of sequences as well as amino acid composition. The second is a large set of 52 894 non-redundant human proteins that do not have any annotation in UniProt for words including ‘allergen’. At low k-mer length both the true and the two false sets give predictions for all input proteins while the predictive power increases with a k-mer length six and the excess of true over false detections remains stable (Fig. 2B). The fact that both negative set curves are very similar shows that, for k-mer studies, even the unnatural but simple to obtain sequence reversal may be a reasonable estimate for false positives. This is supported by studies showing that only in very limited cases; reversed sequences would also represent similar structures (Carugo, 2010). Next, the effect of different k-mer based methods on the excess of % true minus false positives or, in other words, the distance of the true from the false curve from the first graph, was examined (Fig. 2C). The methods included: (i) classical single k-mer hit required for prediction as allergen, (ii) at least three k-mer hits in same sequence for prediction as allergen, (iii) single hit criterion but only if k-mer is not a simple repeat motif as measured by a minimum sequence entropy threshold and (iv) triple hit plus entropy criterion. At k-mer length six, the combined triple hit and entropy criterion performs best (Fig. 2C) and is used in AllerCatPro (Fig. 1, S5). Depending on future extensions of allergen databases these criteria will have to be revisited.

### 3.3 Gluten-like Q-repeats

Filtering out peptides of low complexity to reduce random hits also prevent hits to Gluten-like repeats of Glutamine (Q-repeats), which are important to be recognized especially for immune reactions such as Celiac disease (Hischenhuber *et al.*, 2006; Mamone *et al.*, 2011). To distinguish random hits from Gluten-like Q-repeats and account for its relevance for allergenicity risk assessment, a dedicated score was created based on the compositional fingerprint (Supplementary Fig. S2A) of peptides associated with Celiac disease in the FARRP database (Goodman *et al.*, 2016).

Composition and physical property-based fingerprints have already been used for allergen assessments (Dimitrov *et al.*, 2014b) and we believe this approach to be especially useful for short repeats of the same set of amino acids characters but in different order and combination. Indeed, this compositional fingerprint score significantly separates peptides associated with Celiac disease (‘Celiac peptides’) from human non-allergen peptides (Supplementary Fig. S2B) and detects all known proteins associated with Celiac disease in the database via their short repeats and is therefore included in the AllerCatPro prediction method (Fig. 1, S1 and D3).

### 3.4 Moving from a linear sequence window to 3D epitope similarity

**Fig. 3.**
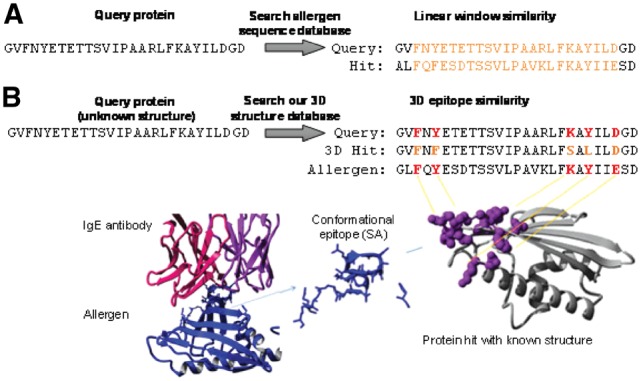
Prediction of linear sequence window and 3D epitope similarity. Screening for similarity between a query protein sequence and a sequence in the allergen database based on a sequence window of 80 residues with at least 35% identity **(A)**. Matching of a query protein sequence with unknown 3D structure and the closest known allergen over all possible 3D structural epitopes within the created comprehensive 3D structural database of known allergens **(B)**

The second traditional criterion that has been suggested for allergenicity assessment is that if a protein matches a known allergen over a linear window of 80 residues with at least 35% identity (FAO/WHO, 2001), then it is declared a potential allergen (Fig. 3A). The rationale behind this criterion is that at this level of similarity the 3D structure of the region may be identical, at least at the domain fold level, which could lead to similar recognition by B cells and IgE antibodies. However, the fast linear-window approach also has its limitations as it ignores the fact that antibody recognition of especially discontinuous epitopes occurs in 3D and surfaces can also differ among same folds. At the same time there have been good efforts to predict 3D cross-reactivity by comparing 3D structures (Ivanciuc *et al.*, 2003; Negi and Braun, 2017). However, 3D structures are known for only a fraction of allergens and it remains a challenge to evaluate a query protein of unknown structure in a fast and automated manner.

To overcome this, AllerCatPro utilizes (i) comprehensive structure modeling to create a 3D database of all known allergens, (ii) a fast sequence-to-structure alignment method and finally (iii) a structural epitope-sized 3D window sliding over the structure to move from the previous linear sequence window approach into relevant 3D structure comparisons (Fig. 3B). To create a comprehensive 3D structural database of known allergens, the allergens were clustered into groups with at least 70% sequence identity and the best templates for structural modeling were predicted for every cluster representative (see Section 2). This clustering avoids overrepresentation of fold members and negative effects from potential modeling inaccuracies of highly similar sequences while still providing efficient coverage of the fold space among all allergens for fast comparison. Crystal structures were considered when available and highly reliable homology models built utilizing methods successfully employed previously for protein structure prediction (Kraft *et al.*, 2005; Kunze *et al.*, 2011; Maurer-Stroh *et al.*, 2003, 2009). With this approach, structures covering 74% of the 4180 known allergens were identified and modeled for AllerCatPro (Fig. 1, D2, see Section 2 for details). Furthermore, we compare the fold classification and distribution of our models to a recent review of known structures of allergens (Dall’antonia *et al.*, 2014) as well as the crystal structures listed at the dedicated Structural Database of Allergenic Proteins (Ivanciuc *et al.*, 2003). We observed that the relative ratios of the major fold classes like all alpha, all beta etc. are maintained but that these bigger classes are proportionally larger than the group of small proteins and peptides (Supplementary Fig. S6).

The next task was to solve the problem of a fast and-automated structure-based comparison for query proteins of unknown structure. For every representative structure in our database, we first pre-calculated all possible epitope-sized 3D surface regions and then created structure-to-sequence maps assigning sequence positions to their respective epitopes. This allows quick recall of sequence-to-structure mappings from sequence-based alignments against the known structures. Therefore, sequence similarity can be used to identify for a query protein sequence both the closest known allergen and the closest structure representative and create a multiple sequence alignment of the three. And finally, using the sequence-to-structure maps of the epitopes, the similarity of the query and the closest known allergen over all possible 3D structural epitopes for the closest 3D surface match can be evaluated (Fig. 3B).

The initial 70% clustering is essential for our epitope approach, since we do not measure explicit 3D structural differences between crystal structures or models but we compare differences in the sequence alignment of a query with the closest known allergen in the context of 3D epitope residues over the same common family structure/model scaffold. If redundancy were to be retained it would create a mix of highly similar structures/models with small and not necessarily reliably modeled conformational fluctuations adding bias to the comparison.

It should be noted that we are not directly considering known B-cell epitopes at this stage because it is difficult to avoid bias towards the set of very well-studied allergens with complete experimental epitope data that is not available for the majority of other allergens. We focused here on a general approach that can be equally used on known and new proteins for the purpose of risk assessment. To exemplify that our epitope comparison includes relevant epitopes also without explicit bias towards experimentally known sites, we show an example of a rice Bet v 1 sequence being compared with known allergens on the well-studied dominant epitope also seen in crystal structures (Supplementary Fig. S7). In this case, the well-known dominant epitope is also the most similar epitope for this sequence but it only has 80% identity in the epitope consistent with expected lower allergenicity potential of the rice Bet v 1.

### 3.5 Combined workflow

Finally, the discussed methods and scores were combined into a decision workflow (Fig. 1A) that is guided by consistency between previous rules and recommendations. The input is a query protein and the output is the model’s assessment if there is strong, weak or no evidence of allergenicity potential for the queried protein based on the different measures of similarity to known allergens. Presence of a Gluten-like Q-repeat is classified as strong evidence independent of other features and, hence, evaluated first. Next, sequence similarity to representatives in our 3D structure database is checked. If there is significant sequence similarity, then the 3D surface epitope similarity is used to assign ‘strong evidence’ if above the benchmark threshold of 93% sequence identity or ‘weak evidence’ otherwise. The rationale here is that sharing the same fold is at least weak evidence for allergenicity potential, but only if surface epitopes are substantially similar one would expect cross-reactivity and hence strong evidence for allergenicity potential. The threshold was chosen to allow correct prediction of all known allergens and thereby maintain highest sensitivity (see benchmark below). If no structure hit is found (as is the case for ∼26% of known allergens), a default back to the classical linear-window approach is used with the established 35% identity over 80 residues rule also resulting in a strong evidence call. Finally, if also no hit was found with the linear-window approach, the model falls back to evaluating by k-mer. This hierarchical staggering also ensures that the more relevant 3D and linear windows are given priority over the k-mers in the evaluation. Only, if none of the methods give a hit, a ‘no evidence’ prediction is assigned. The complete workflow was named ‘AllerCatPro’.

### 3.6 Performance benchmark

Since the aim of this work is to have reliable safety assessments, the thresholds, such as the 3D epitope % identity, have been set to be able to give hits to all known allergens in our database. It needs to be noted, however, that among the 4180 there were 11 sequences (all <18 residues length) that were missed because they are too short to be evaluated (length 5) or have ambiguous characters and do not trigger the model’s k-mer rule. The formal sensitivity is therefore 99.7%. This must be seen as an upper boundary since the sequences to be predicted are also in the AllerCatPro database. To estimate performance on new sequences, first a jack-knife cross-validation was performed where the sequence to be predicted is removed from the database (Supplementary Fig. S3). This still yields 97.2% sensitivity. Extending the cross-validation to removing all sequences with >90% identity produces 93.8% sensitivity. The latter is a common scenario where remote family members of known allergen protein families from other species are being evaluated. It is important to point out that this type of cross-validation is more stringent and systematic than 5- or 10-fold cross-validation with random assignment to groups since it makes sure that no closely related family member remains in the respective ‘training’ sets. Additionally, the sensitivity of the method was also evaluated on previous benchmark sets provided by other tools and it ranges from 96.5 to 99.3% (Supplementary Fig. S4).

To test the performance of the new 3D structure similarity measure in detail, a benchmark set of 221 known allergens with structures in our database was created (selected to be structurally non-redundant using CLICK; Nguyen *et al.*, 2011) and matched with 221 likely non-allergens with the same fold by finding the closest non-self hits in species like human, rice, yeast or E. coli (Supplementary Table S2). We emphasize that this set is small but well representative of protein allergens with structure in our set.

**Fig. 4:**
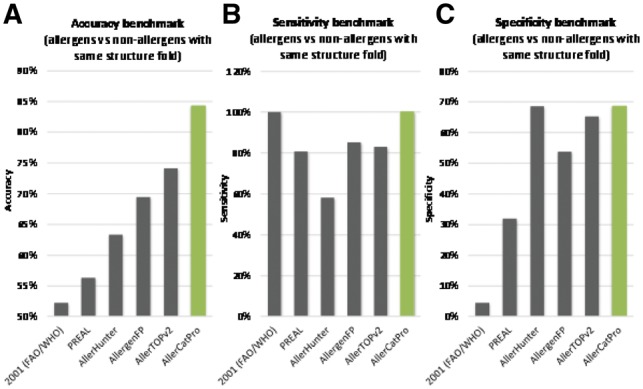
AllerCatPro performance. Performance of AllerCatPro is calculated as accuracy to predict allergens (*n* = 221) versus non-allergens (*n* = 221) with the same structural fold compared with FAO/WHO rules (window-rule only, no k-mer), PREAL, AllerHunter, AllergenFP and AllerTOPv2 **(A)**. By our definition, sharing the fold with an allergen already results in a weak evidence prediction. Therefore, the calculation of accuracy here is based on strong prediction on known allergen as true positive, weak prediction on known allergen as false negative, weak prediction on non-allergen as true negative and strong prediction on non-allergen as false positive. For the same benchmark, the respective sensitivity **(B)** and specificity **(C)** is highlighted

AllerCatPro achieves 84% overall accuracy (Fig. 4A) at 100% sensitivity (Fig. 4B) and 67% specificity (Fig. 4C). From other methods reported in the recent literature results were generated for the same data set, including the classical FAO/WHO linear-window rule (FAO/WHO, 2001) but leaving out the ambiguous k-mer rule that would predict 100% of positives and negatives, PREAL (Wang *et al.*, 2013), AllerHunter (Muh *et al.*, 2009), AllergenFP (Dimitrov *et al.*, 2014b) and AllerTOPv2 (Dimitrov *et al.*, 2014a). The accuracy of these methods ranges from 51% for the old FAO/WHO rules to respectable 73% (AllerTOPv2) (Fig. 4A). The same trends are seen when evaluating by Matthew Correlation Coefficient (Supplementary Fig. S5). However, only the FAO/WHO window rule and AllerCatPro achieve 100% sensitivity (safety rationale for conservative assessments) with the other methods typically ranging from 57 (AllerHunter) to 85% (AllergenFP) (Fig. 4B). When compared with the FAO/WHO window rule, AllerCatPro identified 3-fold less false positives resulting in a 37-fold increase in specificity (Fig. 4C) at the same high sensitivity.

### 3.7 Implementation as webserver

**Fig. 5:**
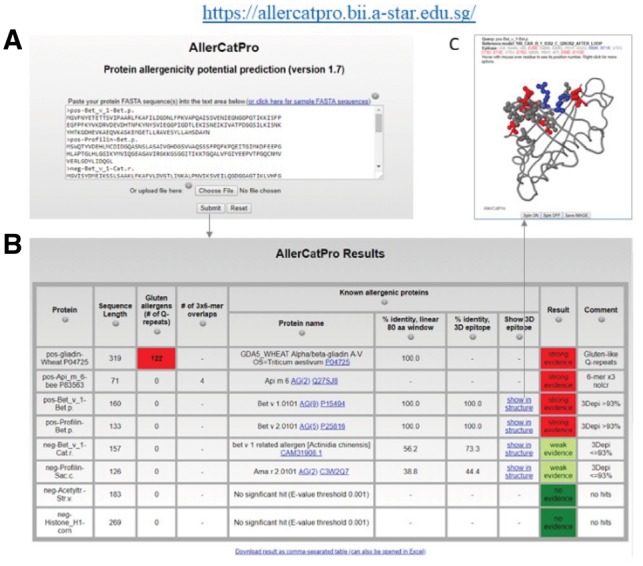
Interface of AllerCatPro version 1.7. Submitting one or more protein sequences in FASTA format **(A)** leads to the AllerCatPro output table with the result for strong, weak or no evidence for allergenicity per protein based on corresponding workflow decisions and, in case of a hit, the possibility to view the most similar proteins **(B)** as well as the most similar 3D surface epitope via links **(C)**. The structural view shows identical epitope residues as balls (colored as blue for positive charges, red for negative charges and gray for all other amino acid types)

AllerCatPro is accessible as a webserver (https://allercatpro.bii.a-star.edu.sg/). The input (Fig. 5A) is one or more protein sequences (up to 50) in FASTA format and the output is a table with the workflow results and decision for one protein per line (Fig. 5B). The results also include a link to view the most similar 3D surface epitope (Fig. 5C) when applicable. At the end there is a download link for the results also in comma-separated format which can be opened by popular spreadsheet programs.

## 4 Conclusions

In this work, we build on and extend the work by several groups and expert panels with the aim to improve assessment of the allergenic potential of protein sequences. Our emphasis has been to retain earlier considerations and update or upgrade the approach and criteria. Starting with a comprehensive database comparison to derive the largest set of reliable known allergens, we propose an entropy-adjusted hexamer hit approach as well as switching from linear sequence window similarity to B-cell epitope-like 3D surface similarity with predicted structures for 74% of all known allergens in a workflow guided by safety rationale. At the highest sensitivity needed for conservative assessments, AllerCatPro increases specificity by 37-fold compared with the previous rules.

## Supplementary Material

btz029_Supplementary_Figures-Tables

## References

[btz029-B1] Altschul S.F. et al (1997) Gapped BLAST and PSI-BLAST: a new generation of protein database search programs. Nucleic Acids Res., 25, 3389–3402. 9254694 10.1093/nar/25.17.3389 PMC146917

[btz029-B2] Burley S.K. et al (2017) Protein Data Bank (PDB): the single global macromolecular structure archive. Methods Mol. Biol., 1607, 627–641. 28573592 10.1007/978-1-4939-7000-1_26 PMC5823500

[btz029-B3] Carugo O. (2010) Structural similarity between native proteins and chimera constructs obtained by inverting the amino acid sequence. Acta Chim Slov., 57, 936–940. 24061900

[btz029-B4] Dall’antonia F. et al (2014) Structure of allergens and structure based epitope predictions. Methods, 66, 3–21. 23891546 10.1016/j.ymeth.2013.07.024 PMC3969231

[btz029-B5] Dimitrov I. et al (2014a) AllerTOP v.2–a server for in silico prediction of allergens. J. Mol. Model., 20, 2278. 24878803 10.1007/s00894-014-2278-5

[btz029-B6] Dimitrov I. et al (2014b) AllergenFP: allergenicity prediction by descriptor fingerprints. Bioinformatics, 30, 846–851. 24167156 10.1093/bioinformatics/btt619

[btz029-B7] FAO/WHO. (2001) Evaluation of Allergenicity of Genetically Modified Foods: Report of a Joint FAO/WHO Expert Consultation on Allergenicity of Foods Derived from Biotechnology. Food and Agriculture Organization of the United Nations (FAO) and World Health Organization (WHO), Rome, Italy, pp. 22–25.

[btz029-B8] Fukutomi Y. et al (2014) Epidemiological link between wheat allergy and exposure to hydrolyzed wheat protein in facial soap. Allergy, 69, 1405–1411. 25040662 10.1111/all.12481

[btz029-B9] Goodman R.E. et al (2016) AllergenOnline: a peer-reviewed, curated allergen database to assess novel food proteins for potential cross-reactivity. Mol. Nutr. Food Res., 60, 1183–1198. 26887584 10.1002/mnfr.201500769

[btz029-B10] Goodman R.E. (2006) Practical and predictive bioinformatics methods for the identification of potentially cross-reactive protein matches. Mol. Nutr. Food Res., 50, 655–660. 16810734 10.1002/mnfr.200500277

[btz029-B11] Herman R.A. et al (2009) Value of eight-amino-acid matches in predicting the allergenicity status of proteins: an empirical bioinformatic investigation. Clin. Mol. Allergy, 7, 9. 19874602 10.1186/1476-7961-7-9 PMC2773230

[btz029-B12] Hileman R.E. et al (2002) Bioinformatic methods for allergenicity assessment using a comprehensive allergen database. Int. Arch. Allergy Immunol., 128, 280–291. 12218366 10.1159/000063861

[btz029-B13] Hischenhuber C. et al (2006) Review article: safe amounts of gluten for patients with wheat allergy or coeliac disease. Aliment. Pharmacol. Ther., 23, 559–575. 16480395 10.1111/j.1365-2036.2006.02768.x

[btz029-B14] Huby R.D. et al (2000) Why are some proteins allergens? Toxicol. Sci., 55, 235–246. 10828254 10.1093/toxsci/55.2.235

[btz029-B15] Ivanciuc O. et al (2003) SDAP: database and computational tools for allergenic proteins. Nucleic Acids Res., 31, 359–362. 12520022 10.1093/nar/gkg010 PMC165457

[btz029-B16] Jahn-Schmid B. et al (2005) Bet v 1142-156 is the dominant T-cell epitope of the major birch pollen allergen and important for cross-reactivity with Bet v 1-related food allergens. J. Allergy Clin. Immunol., 116, 213–219. 15990797 10.1016/j.jaci.2005.04.019

[btz029-B17] Katoh K., Standley D.M. (2014) MAFFT: iterative refinement and additional methods. Methods Mol. Biol., 1079, 131–146. 24170399 10.1007/978-1-62703-646-7_8

[btz029-B18] Kraft C. et al (2005) The WD40 propeller domain of Cdh1 functions as a destruction box receptor for APC/C substrates. Mol. Cell, 18, 543–553. 15916961 10.1016/j.molcel.2005.04.023

[btz029-B19] Krieger E. et al (2009) Improving physical realism, stereochemistry, and side-chain accuracy in homology modeling: four approaches that performed well in CASP8. Proteins, 77 (Suppl. 9), 114–122. 19768677 10.1002/prot.22570 PMC2922016

[btz029-B20] Krieger E., Vriend G. (2014) YASARA View—molecular graphics for all devices—from smartphones to workstations. Bioinformatics, 30, 2981–2982. 24996895 10.1093/bioinformatics/btu426 PMC4184264

[btz029-B21] Kunze M. et al (2011) Structural requirements for interaction of peroxisomal targeting signal 2 and its receptor PEX7. J. Biol. Chem., 286, 45048–45062. 22057399 10.1074/jbc.M111.301853 PMC3247985

[btz029-B22] Li W., Godzik A. (2006) Cd-hit: a fast program for clustering and comparing large sets of protein or nucleotide sequences. Bioinformatics, 22, 1658–1659. 16731699 10.1093/bioinformatics/btl158

[btz029-B23] Lippert R.A. et al (2002) Distributional regimes for the number of k-word matches between two random sequences. Proc. Natl. Acad. Sci. USA, 99, 13980–13989. 12374863 10.1073/pnas.202468099 PMC137823

[btz029-B24] Mamone G. et al (2011) Proteomic analysis in allergy and intolerance to wheat products. Expert Rev. Proteomics, 8, 95–115. 21329430 10.1586/epr.10.98

[btz029-B25] Maurer-Stroh S. et al (2009) Mapping the sequence mutations of the 2009 H1N1 influenza A virus neuraminidase relative to drug and antibody binding sites. Biol. Direct., 4, 18. discussion 18. 19457254 10.1186/1745-6150-4-18 PMC2691737

[btz029-B26] Maurer-Stroh S. et al (2003) The Tudor domain ‘Royal Family’: Tudor, plant Agenet, Chromo, PWWP and MBT domains. Trends Biochem. Sci., 28, 69–74. 12575993 10.1016/S0968-0004(03)00004-5

[btz029-B27] Muh H.C. et al (2009) AllerHunter: a SVM-pairwise system for assessment of allergenicity and allergic cross-reactivity in proteins. PLoS One, 4, e5861. 19516900 10.1371/journal.pone.0005861 PMC2689655

[btz029-B28] Negi S.S., Braun W. (2017) Cross-React: a new structural bioinformatics method for predicting allergen cross-reactivity. Bioinformatics, 33, 1014–1020. 28062447 10.1093/bioinformatics/btw767 PMC5860227

[btz029-B29] Nguyen M.N. et al (2011) CLICK–topology-independent comparison of biomolecular 3D structures. Nucleic Acids Res., 39, W24–W28. 21602266 10.1093/nar/gkr393 PMC3125785

[btz029-B30] O’Brien R.M. et al (1995) An immunogenetic analysis of the T-cell recognition of the major house dust mite allergen Der p 2: identification of high- and low-responder HLA-DQ alleles and localization of T-cell epitopes. Immunology, 86, 176–182. 7490115 PMC1383992

[btz029-B31] Oseroff C. et al (2012) Analysis of T cell responses to the major allergens from German cockroach: epitope specificity and relationship to IgE production. J. Immunol., 189, 679–688. 22706084 10.4049/jimmunol.1200694 PMC3392449

[btz029-B32] Pawankar R. et al (2013) White Book on Allergy. Update 2013 World Allergy Organization (WAO) Milwaukee, Wisconsin.

[btz029-B33] Pomes A. et al (2018) WHO/IUIS allergen nomenclature: providing a common language. Mol. Immunol., 100, 3–13. 29625844 10.1016/j.molimm.2018.03.003 PMC6019191

[btz029-B34] Prickett S.R. et al (2015) Immunoregulatory T cell epitope peptides: the new frontier in allergy therapy. Clin. Exp. Allergy, 45, 1015–1026. 25900315 10.1111/cea.12554 PMC4654246

[btz029-B35] Ray A. et al (2012) Improved model quality assessment using ProQ2. BMC Bioinformatics, 13, 224. 22963006 10.1186/1471-2105-13-224 PMC3584948

[btz029-B36] Stadler M.B., Stadler B.M. (2003) Allergenicity prediction by protein sequence. FASEB J., 17, 1141–1143. 12709401 10.1096/fj.02-1052fje

[btz029-B37] Tan J. et al (2012) Tachyon search speeds up retrieval of similar sequences by several orders of magnitude. Bioinformatics, 28, 1645–1646. 22531216 10.1093/bioinformatics/bts197 PMC3371831

[btz029-B38] Troyano E. et al (2011) Approach to assessing consumer safety of botanical ingredients with emphasis to type I allergy In: Nava,D. and Lambros,K. (eds) Formulating, Packaging, and Marketing of Natural Cosmetic Products. John Wiley & Sons, Hoboken, New Jersey, US, pp. 149–167.

[btz029-B39] Verhoeckx K. et al (2016) Allergenicity assessment strategy for novel food proteins and protein sources. Regul. Toxicol. Pharmacol., 79, 118–124. 27012375 10.1016/j.yrtph.2016.03.016

[btz029-B40] Wang J. et al (2013) PREAL: prediction of allergenic protein by maximum Relevance Minimum Redundancy (mRMR) feature selection. BMC Syst. Biol., 7 (Suppl. 5), S9. 10.1186/1752-0509-7-S5-S9 PMC4029432 24565053

[btz029-B41] Wang L.-P. et al (2017) Building a more predictive protein force field: a systematic and reproducible route to AMBER-FB15. J. Phys. Chem. B, 121, 4023–4039. 28306259 10.1021/acs.jpcb.7b02320 PMC9724927

[btz029-B42] Webb B., Sali A. (2017) Protein structure modeling with MODELLER. Methods Mol. Biol., 1654, 39–54. 28986782 10.1007/978-1-4939-7231-9_4

[btz029-B43] Westernberg L. et al (2016) T-cell epitope conservation across allergen species is a major determinant of immunogenicity. J. Allergy Clin. Immunol., 138, 571–578.e7. 26883464 10.1016/j.jaci.2015.11.034 PMC4975972

[btz029-B44] Wootton J.C., Federhen S. (1996) Analysis of compositionally biased regions in sequence databases. Methods Enzymol., 266, 554–571. 8743706 10.1016/s0076-6879(96)66035-2

[btz029-B45] Zimmermann L. et al (2018) A completely reimplemented mpi bioinformatics toolkit with a new HHpred server at its core. J. Mol. Biol., 430, 2237–2243. 29258817 10.1016/j.jmb.2017.12.007

