# Supplementary material for: AllerCatPro—prediction of protein allergenicity potential from the protein sequence
Source: Bioinformatics. 2019 Jan 18;35(17):3020–7. doi: 10.1093/bioinformatics/btz029 (PMC6736023; doi:10.1093/bioinformatics/btz029)
Supplement: btz029_Supplementary_Figures-Tables [file btz029_supplementary_figures-tables.zip › btz029-suppl_data/AllerCatPro_SupplementaryFigures_revision1812.pptx]

## Slide 1
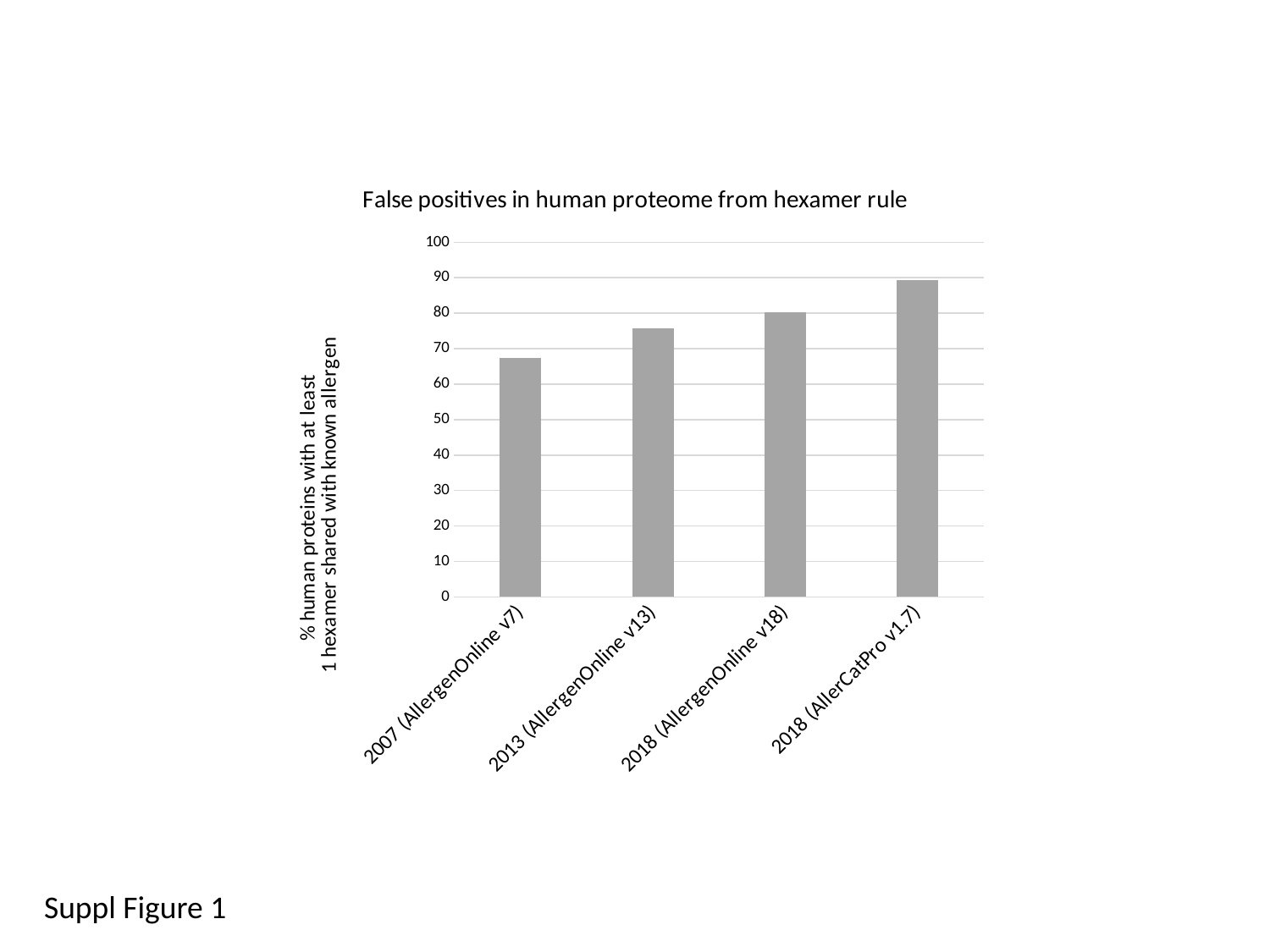

### Chart: False positives in human proteome from hexamer rule
| Category | |
|---|---|
| 2007 (AllergenOnline v7) | 67.348 |
| 2013 (AllergenOnline v13) | 75.661 |
| 2018 (AllergenOnline v18) | 80.279 |
| 2018 (AllerCatPro v1.7) | 89.254 |Suppl Figure 1

## Slide 2
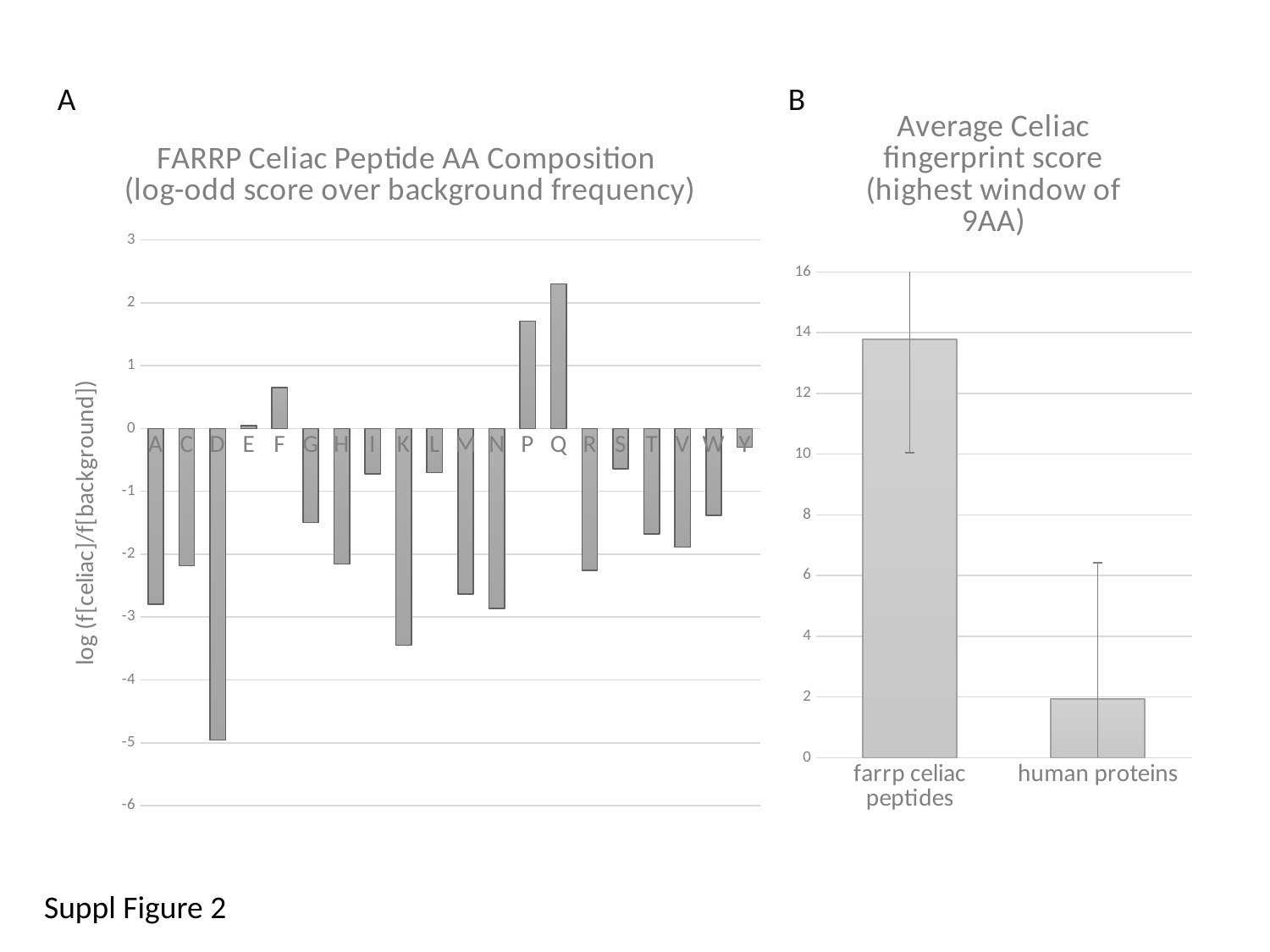

A
B
### Chart: Average Celiac fingerprint score (highest window of 9AA)
| Category | |
|---|---|
| farrp celiac peptides | 13.778887005254003 |
| human proteins | 1.9377656316406375 |
### Chart: FARRP Celiac Peptide AA Composition
(log-odd score over background frequency)
| Category | |
|---|---|
| A | -2.79424 |
| C | -2.18348 |
| D | -4.95254 |
| E | 0.04579 |
| F | 0.64913 |
| G | -1.49454 |
| H | -2.15809 |
| I | -0.72296 |
| K | -3.44772 |
| L | -0.70405 |
| M | -2.63426 |
| N | -2.86356 |
| P | 1.70891 |
| Q | 2.29878 |
| R | -2.25379 |
| S | -0.64406 |
| T | -1.67531 |
| V | -1.88618 |
| W | -1.38276 |
| Y | -0.29713 |Suppl Figure 2

## Slide 3
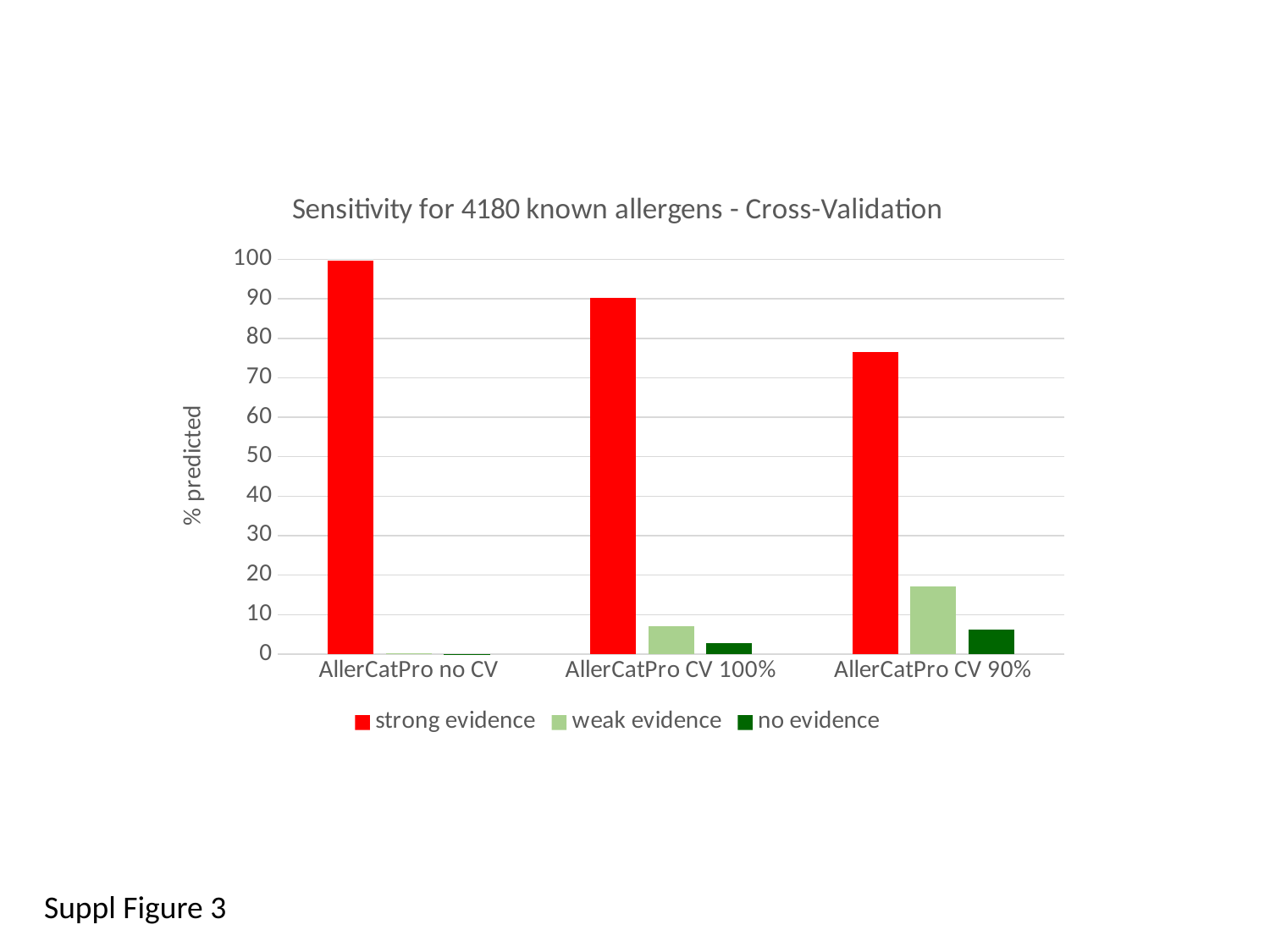

### Chart: Sensitivity for 4180 known allergens - Cross-Validation
| Category | strong evidence | weak evidence | no evidence |
|---|---|---|---|
| AllerCatPro no CV | 99.6 | 0.3 | 0.1 |
| AllerCatPro CV 100% | 90.2 | 7.0 | 2.8 |
| AllerCatPro CV 90% | 76.5 | 17.2 | 6.2 |Suppl Figure 3

## Slide 4
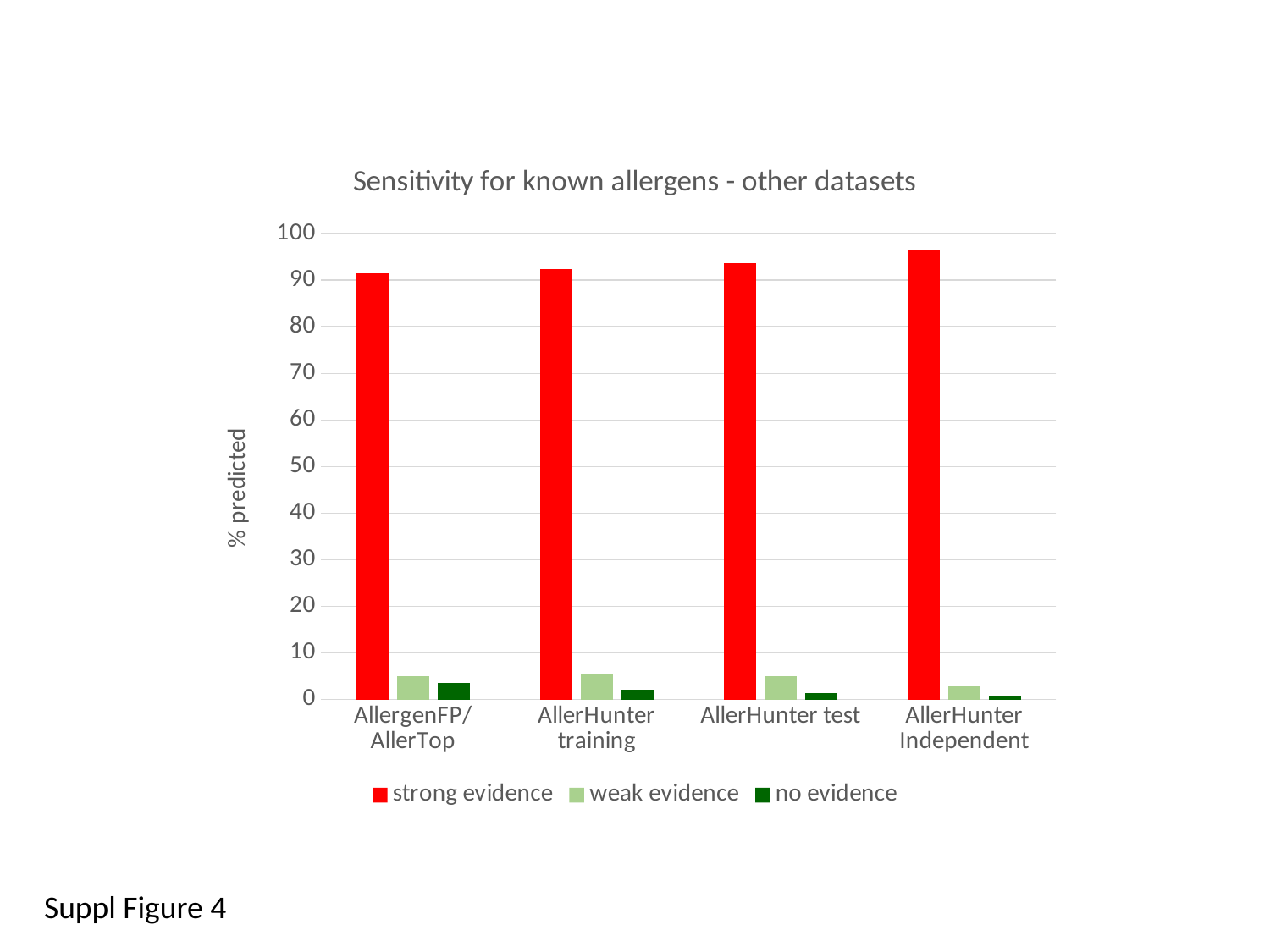

### Chart: Sensitivity for known allergens - other datasets
| Category | strong evidence | weak evidence | no evidence |
|---|---|---|---|
| AllergenFP/AllerTop | 91.4 | 5.1 | 3.5 |
| AllerHunter training | 92.4 | 5.4 | 2.2 |
| AllerHunter test | 93.6 | 5.0 | 1.4 |
| AllerHunter Independent | 96.4 | 2.9 | 0.7 |Suppl Figure 4

## Slide 5
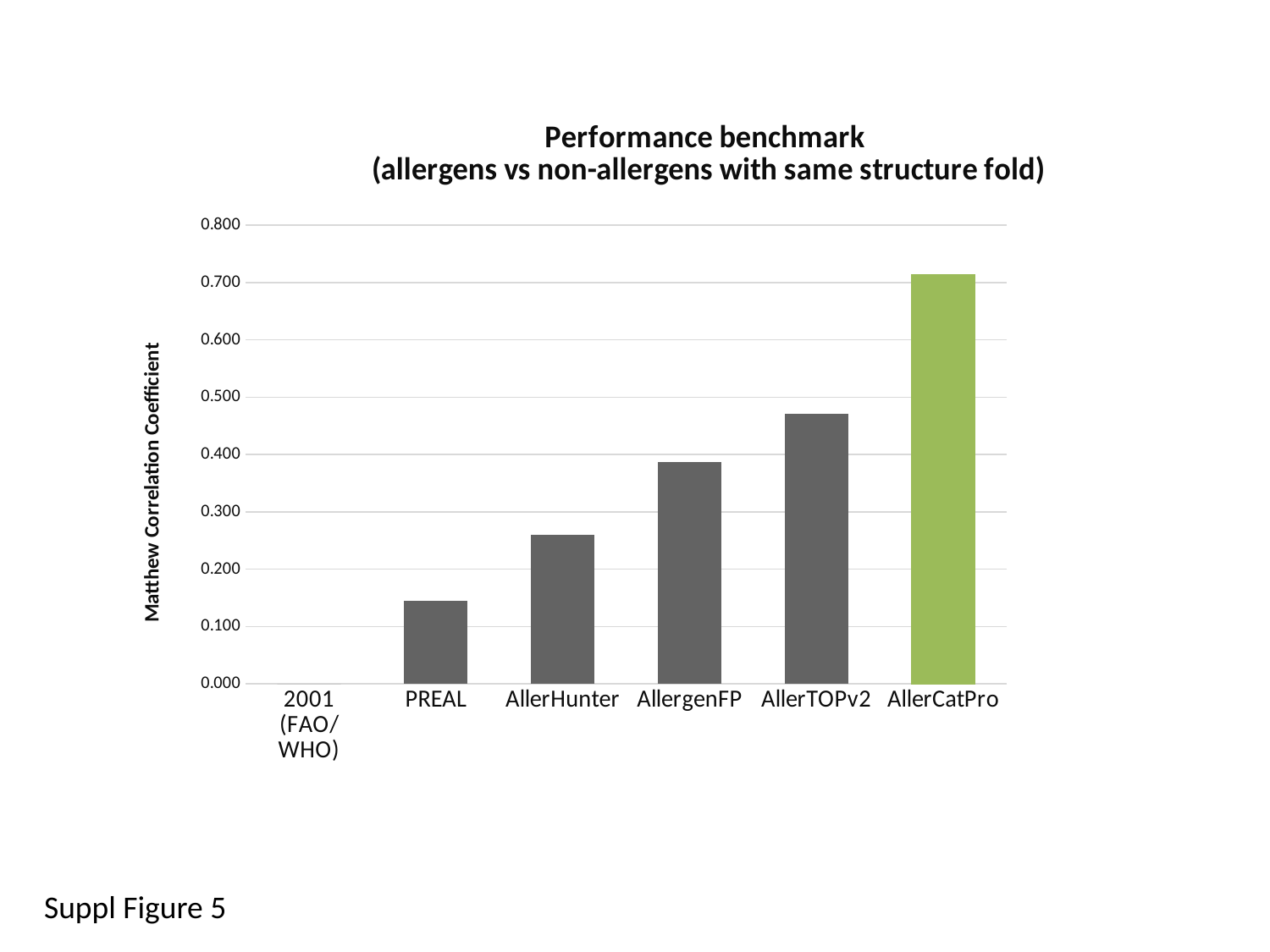

### Chart: Performance benchmark
(allergens vs non-allergens with same structure fold)
| Category | |
|---|---|
| 2001 (FAO/WHO) | 0.0 |
| PREAL | 0.14518704881564082 |
| AllerHunter | 0.25972220024821746 |
| AllergenFP | 0.3871150772553619 |
| AllerTOPv2 | 0.4710463753916303 |
| AllerCatPro | 0.713114593321572 |Suppl Figure 5

## Slide 6
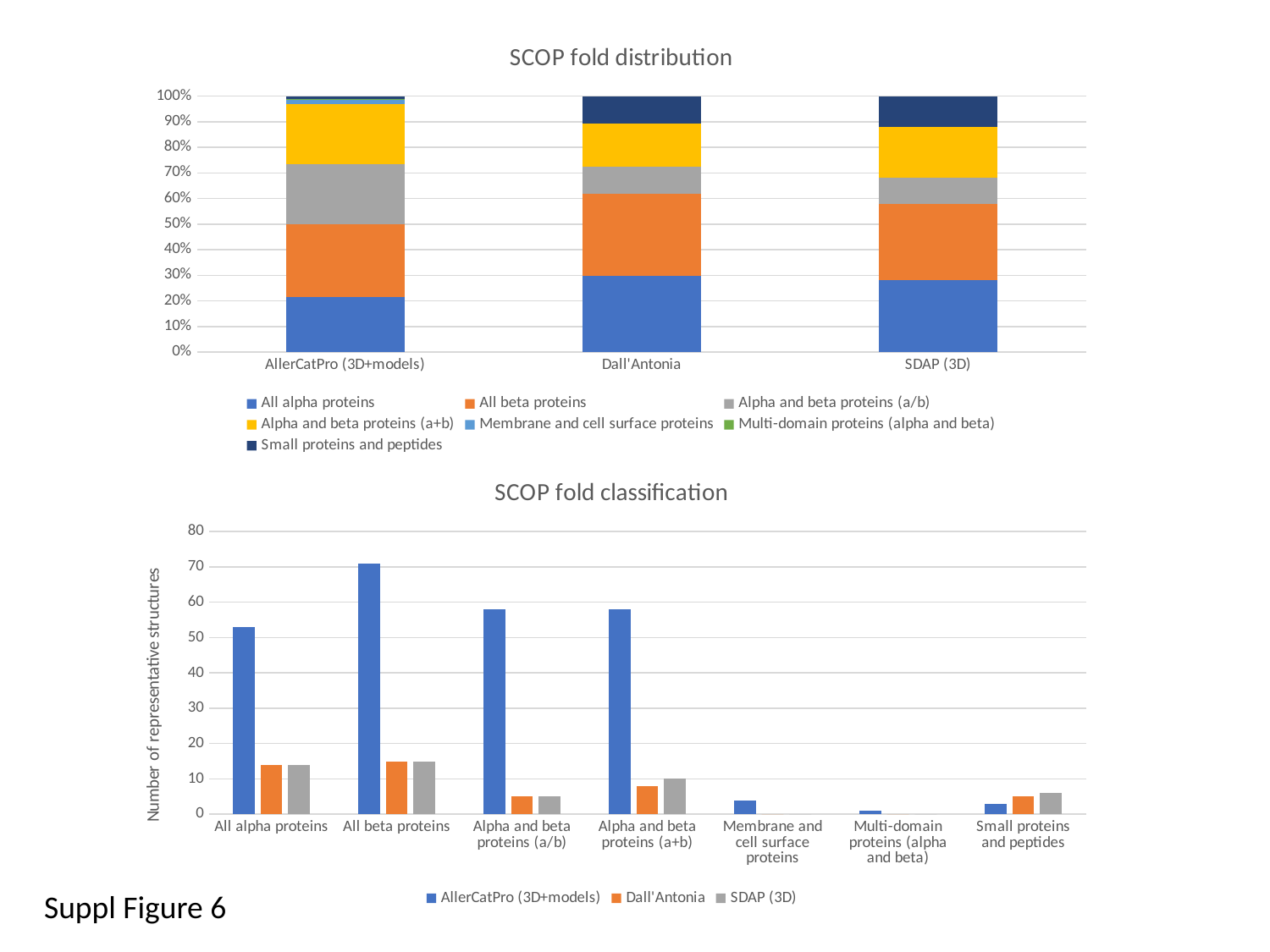

### Chart: SCOP fold distribution
| Category | All alpha proteins | All beta proteins | Alpha and beta proteins (a/b) | Alpha and beta proteins (a+b) | Membrane and cell surface proteins | Multi-domain proteins (alpha and beta) | Small proteins and peptides |
|---|---|---|---|---|---|---|---|
| AllerCatPro (3D+models) | 53.0 | 71.0 | 58.0 | 58.0 | 4.0 | 1.0 | 3.0 |
| Dall'Antonia | 14.0 | 15.0 | 5.0 | 8.0 | 0.0 | 0.0 | 5.0 |
| SDAP (3D) | 14.0 | 15.0 | 5.0 | 10.0 | 0.0 | 0.0 | 6.0 |
### Chart: SCOP fold classification
| Category | AllerCatPro (3D+models) | Dall'Antonia | SDAP (3D) |
|---|---|---|---|
| All alpha proteins | 53.0 | 14.0 | 14.0 |
| All beta proteins | 71.0 | 15.0 | 15.0 |
| Alpha and beta proteins (a/b) | 58.0 | 5.0 | 5.0 |
| Alpha and beta proteins (a+b) | 58.0 | 8.0 | 10.0 |
| Membrane and cell surface proteins | 4.0 | 0.0 | 0.0 |
| Multi-domain proteins (alpha and beta) | 1.0 | 0.0 | 0.0 |
| Small proteins and peptides | 3.0 | 5.0 | 6.0 |Suppl Figure 6

## Slide 7
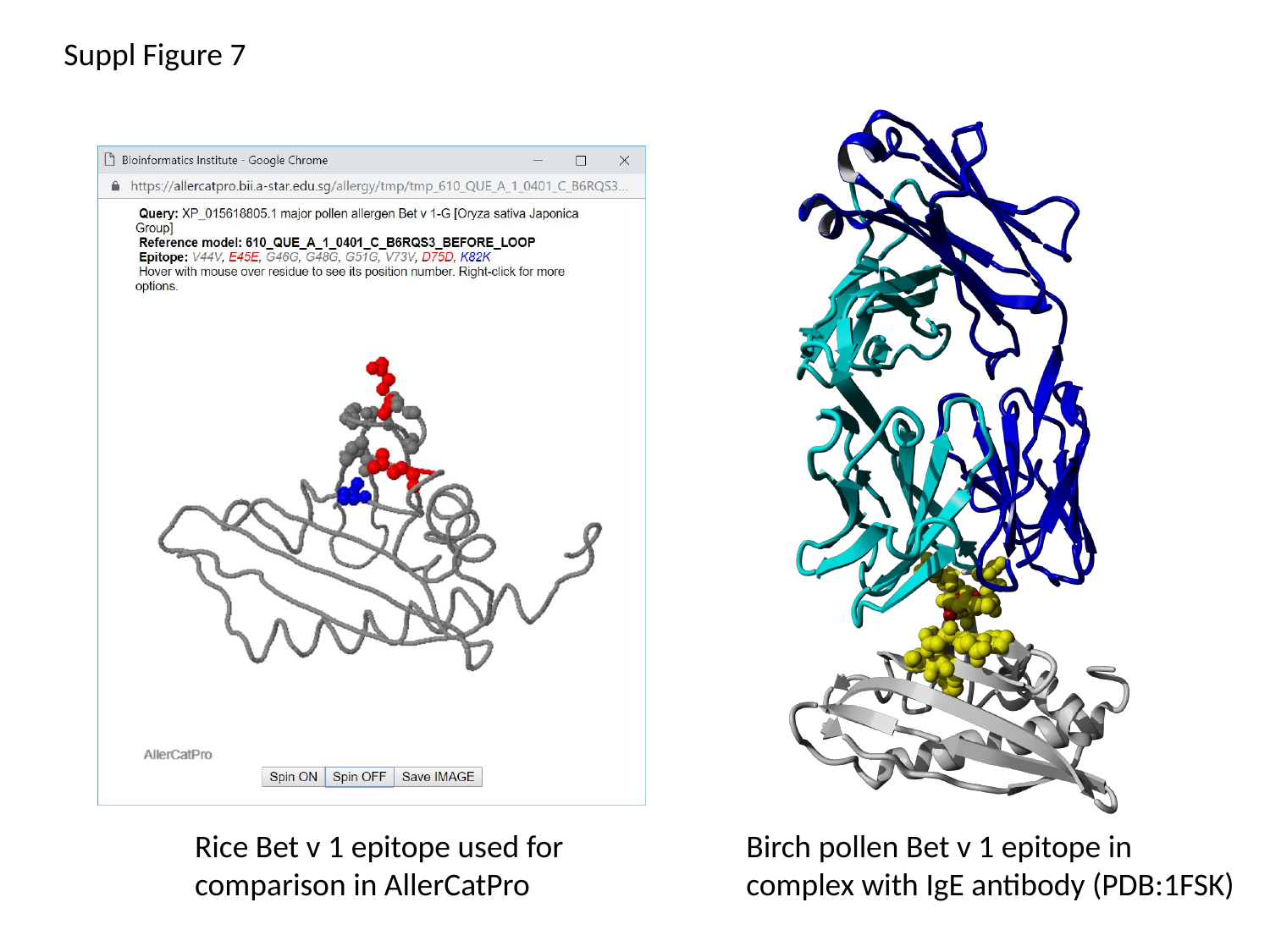

Suppl Figure 7
Birch pollen Bet v 1 epitope in complex with IgE antibody (PDB:1FSK)
Rice Bet v 1 epitope used for comparison in AllerCatPro
